# The influence of banner advertisements on attention and memory: human faces with averted gaze can enhance advertising effectiveness

**DOI:** 10.3389/fpsyg.2014.00166

**Published:** 2014-03-04

**Authors:** Pitch Sajjacholapunt, Linden J. Ball

**Affiliations:** ^1^Department of Psychology, Lancaster UniversityLancaster, UK; ^2^School of Psychology, University of Central LancashirePreston, UK

**Keywords:** online banner advertising, human faces, eye tracking, gaze cues, averted gaze, mutual gaze, memory, advertising effectiveness

## Abstract

Research suggests that banner advertisements used in online marketing are often overlooked, especially when positioned horizontally on webpages. Such inattention invariably gives rise to an inability to remember advertising brands and messages, undermining the effectiveness of this marketing method. Recent interest has focused on whether human faces within banner advertisements can increase attention to the information they contain, since the gaze cues conveyed by faces can influence where observers look. We report an experiment that investigated the efficacy of faces located in banner advertisements to enhance the attentional processing and memorability of banner contents. We tracked participants' eye movements when they examined webpages containing either bottom-right vertical banners or bottom-center horizontal banners. We also manipulated facial information such that banners either contained no face, a face with mutual gaze or a face with averted gaze. We additionally assessed people's memories for brands and advertising messages. Results indicated that relative to other conditions, the condition involving faces with averted gaze increased attention to the banner overall, as well as to the advertising text and product. Memorability of the brand and advertising message was also enhanced. Conversely, in the condition involving faces with mutual gaze, the focus of attention was localized more on the face region rather than on the text or product, weakening any memory benefits for the brand and advertising message. This detrimental impact of mutual gaze on attention to advertised products was especially marked for vertical banners. These results demonstrate that the inclusion of human faces with averted gaze in banner advertisements provides a promising means for marketers to increase the attention paid to such adverts, thereby enhancing memory for advertising information.

## Introduction

The Internet World Statistic Report (2012) indicated that nearly 2 billion people were using the Internet in 2011, compared to 360 million in 2000. This major growth in Internet usage has been paralleled by an exponential increase in online advertising, with investment reaching an estimated 31 billion dollars in 2011, surpassing that of advertising via cable and broadcast television (Internet Advertising Revenue Report, 2012). Website advertisements include pop-ups, videos and on-site sponsorship (Schumann and Thorson, 2007), but it is the simple banner advertisement that appears to be the most enduring format subsequent to its initial appearance in 1994 (Cho, 2003). Banner advertisements arise in various rectangular-shaped graphics, including skyscrapers (120 × 600 pixels), squares (250 × 250 pixels), large rectangles (336 × 280 pixels) and vertical rectangles (240 × 400 pixels). Color, animation, and interactivity are often included in the advertisement in an attempt to capture attention, with the interactivity element also providing a way to track user interest (Zeff and Aronson, 1999). Ultimately, the popularity of banner advertisements appears to derive from their considerable flexibility and targetability as devices for marketing products and brands.

Despite the dominance of banner advertisements in Internet advertising, their effectiveness remains debatable. Benway and Lane (1998) demonstrated that web users tend to avoid looking at such advertisements even when they are designed to be attention-grabbing—a phenomenon referred to as “banner blindness.” More recent research has emphasized the importance of quantifying the effectiveness of banner advertisements: (1) by using metrics derived from eye-movement tracking, which can indicate overt attentional shifts to such advertisements; and (2) through tests of people's memory for banner contents. Using such measures, Drèze and Hussherr (2003) showed that web users fixate more on banner advertisements that are relevant to their goal-directed searches, which also leads to an increase in the memorability of those advertisements. They also found that banner advertisements were more effective when placed vertically to the left or right of webpages, as opposed to horizontally at the top or bottom of webpages.

Drèze and Hussherr's (2003) results also resonate with recent findings from a series of eye tracking experiments reported by Simola et al. (2011), which examined the attentional impact of salient advertisements placed simultaneously at two locations on authentic webpages: above a central portion of text and to the right of the text. Results indicated that banner advertisements attracted overt attention (as indexed by eye fixations on the advertisement) and that such attentional capture was especially marked when the advertisements were vertical and to the right of the webpage text. Simola et al. propose that this effect is a likely consequence of Western readers having a perceptual span that is highly biased toward the right of fixation (i.e., around 15 letters) rather than to the left of fixation (only around 3–4 letters; see Rayner, 1998). Simola et al. (2011) also found that right-located vertical banners were particularly attention demanding either when they contained animated features that contrasted with static horizontal advertisements appearing simultaneously at the top of the page or when they appeared abruptly after a random time interval.

The findings arising from the research of Drèze and Hussherr (2003) and Simola et al. (2011) pose a problem for investment in online advertising given that horizontal banners are far more prevalent on websites than vertical banners (Hussain et al., 2010), whereas it is vertical banners located to the right of webpage text that appear to have a greater capacity to capture attention (see Simola et al., 2013, for similar evidence from a study of attention and memory for newspaper advertisements). It is also interesting to reflect on the issue of banner location in the light of Nielsen's (2006) research, which has shown that web users normally extract information from webpages in an F-shaped pattern: they start off looking at page elements from the top left to the top right, they then read down the page slightly, again from left to the right, and finally continue to fixate downward on the left side of the page. This F-shaped reading pattern would suggest that it is should be components that are placed at the bottom center and the bottom right of a webpage that are most likely to be *overlooked* (see also Djamasbi et al., 2010). However, Simola et al.'s (2011) findings raise the possibility that attentional capture to banner advertisements may be effective even for vertical advertisements located to the bottom-right of pages in cases where such advertisements are co-located alongside webpage text. Admittedly, this proposal has not yet received empirical support since Simola et al's experiments only involved vertical banners that extended well above the half-way point of webpages. The present research therefore aimed to address the banner location issue by manipulating the position of banners on webpages such that they appeared either at the bottom-right of presented webpages in a position adjacent to the centrally-located text or at the bottom-center of webpages. According to Nielsen's (2006) F-shaped reading pattern it would be expected that both of these banner locations would be equally poor for attention capture. In contrast, Simola et al.'s (2011) findings lead to the prediction that the vertical banners (located bottom-right) should be associated with increased attentional capture relative to the horizontal banners (located bottom-center).

Clearly, any on-line advertisements that fail to capture or hold a viewer's attention will generally be ineffective in instilling product knowledge or brand awareness (Keller and Lehmann, 2006; Maughan et al., 2007). This is why advertisers have become increasingly interested in ways to augment the attention-grabbing capacity of on-line advertisements using techniques such as animation or their abrupt appearance, which can drive attention in a “bottom-up,” data-driven manner. However, there is also evidence that web users are able to exercise strategic, top-down control of attention such that they can override bottom-up attentional capture arising from salient low-level information such as motion (e.g., see Burke et al., 2005). In addition, there is evidence that having to exercise such top-down control leads web users to report negatively about their website experience, claiming higher perceived workload and a greater sense of irritation and distraction (e.g., Zhang, 2000; Gao et al., 2004; Burke et al., 2005). The negative effects of animated advertisements on the experience of web users means that advertisers are continually examining new and more subtle ways to design banner advertisements that may have a facilitatory impact on people's attention allocation and memory without being annoying. One factor that Wedel and Pieters (2007) suggest needs far greater research in online advertising contexts is the role of the human face, which may be able to draw a viewer's attention to banner advertisements and the content therein. The present research aimed to investigate the capacity of human faces to capture attention to banner advertisements and thereby to facilitate enhanced memory for banner contents. We examined this issue in conjunction with assessing the banner location factor that we have already discussed.

Returning to the potential role of facial images in cueing attention we note that faces are considered to be uniquely potent stimuli for attracting visual attention owing to their social importance for understanding others' characteristics, personalities, intentions, and emotions (e.g., Emery, 2000; Vuilleumier and Schwartz, 2001). Evidence from neuroimaging (e.g., Kanwisher et al., 1997) indicates that face perception is underpinned by specialized neural systems (Tsao and Livingstone, 2008), whilst behavioral data show that when faces are presented in a visual scene along with other stimuli they capture a viewer's attention more readily than do the other objects (Vuilleumier, 2000; Ro et al., 2001). Indeed, Langton et al. (2008) found that when participants were asked to search for a target object (images of butterflies) in the presence of an irrelevant image of a human face they found the face distracting. These findings suggest that faces might well serve as a powerful means for attracting and holding a viewer's attention in an online advertising context.

Eye-movement studies of face processing (e.g., Althoff and Cohen, 1999) have clarified that people spend more time viewing internal features of faces (i.e., the eyes, nose, and mouth) than external features (i.e., hair, ears, and face contours). Indeed, many studies have shown that the eyes are the most attended facial region and are the most valuable source of information for social communication (e.g., through the portrayal of emotion and thought) and for directing the attention of others (e.g., Henderson et al., 2005; Frischen et al., 2007; Itier and Batty, 2009). If the position of the dark iris is observed to be in the middle of the white sclera, then people perceive this gaze as looking straight at them (i.e., “direct” or “mutual” gaze). In contrast, if the position of the dark iris is situated to the left or the right of the sclera, thereby creating the large visible area of white, then the observer perceives this as “averted” gaze (Itier and Batty, 2009).

Research has indicated that mutual gaze is more efficient in capturing attention than averted gaze (e.g., Senju et al., 2005; Conty et al., 2006; Frischen et al., 2007), whilst other studies have shown that averted gaze conveys to an observer that the person being observed (we subsequently use the term “model”) is paying attention to a particular object or location that follows their direction of gaze (Baron-Cohen, 1995; Emery, 2000). Such averted gaze can thereby have an impact on orienting the focus of an observer's attention such that both the model and the observer pay attention to the same location or object and engage in “joint attention” (Itier and Batty, 2009). Evidence also supports the view that the gaze cueing that leads to joint attention arises rapidly and reflexively (e.g., Friesen and Kingstone, 1998; Hood et al., 1998). Although many studies have addressed the reflexive, orienting effects of averted gaze cueing in naturalistic situations with actual people present, there are also numerous studies that have used photographic depictions of a human face presented centrally to an observer (Driver et al., 1999; Langton and Bruce, 1999; Vuilleumier, 2002; Mansfield et al., 2003). This research has again shown reflexive shifts of attention with photographic images depicting averted gaze (see also Ricciardelli et al., 2002; Mansfield et al., 2003), confirming that photographic images of faces can drive a stimulus-driven orienting response toward a gazed-at location that cannot be suppressed.

The aforementioned findings suggest that placing an image of a face within a banner advertisement with the face depicting averted gaze might serve as an effective trigger for capturing and orienting a viewer's attention toward advertised information, despite the presence of other stimuli on the webpage. To examine this possibility the experiment we report below manipulated the presence vs. absence of faces within banner advertisements and also examined the issue of whether mutual gaze vs. averted gaze might differentially impact the level of attention to advertised information. Based on existing evidence we would predict that faces involving mutual gaze would lead viewers to pay more attention to the model's face itself rather than to the text or products in the advertisements. In contrast, faces involving averted gaze would be predicted to orient reflexively the focus of a viewer's attention to advertised texts and products embedded within the advertisements.

Very little research appears to have been undertaken to explore the power of a model's gaze cues to influence people's attention toward print and online advertisements. One relevant study is reported by Straub (2008), who used eye-tracking and the presentation of a female face on a computer screen to examine the effect of gaze cues (mutual gaze vs. averted gaze) on attention to a shampoo advertisement. The results suggested that when the eyes of the model were looking at the advertised text and product (averted gaze), then participants were likely to look at the internal features on the face (e.g., eyes and nose) and then to fixate intensively on the advertised text and products. Conversely, when the eyes of the same model were looking straight ahead at the viewer (mutual gaze), participants were prone to fixate intensely on the face while spending less time on the advertised text and products. The results of this study support the prediction that the perception of different gaze direction can affect the gaze patterns of viewers looking at advertisements on the screen, but it remains unknown what the impact might be of gaze cues on banner advertisements located on webpages involving realistic online content.

An attendant issue that has not been examined concerns the effects of human faces with gaze cues on people's memory for advertisements. Although it is known that information that is fixated for longer tends to be better remembered (Irwin and Zelinsky, 2002), it is nevertheless, important to generalize this finding to the context of banner advertising. As such, the present research not only addressed the influence of gaze cues on attention to banner advertisements but also the effectiveness of these gaze cues on memory for advertising content. Many eye-tracking studies have shown that memory for advertised text or brands contained in banner advertisements is poor, even when the banner advertisement has been fixated, although there is also evidence that memory for banner contents is positively correlated with the overall time that people attend to the advertisement (e.g., Drèze and Hussherr, 2003; Burke et al., 2005).

It should be noted, however, that most studies of memory for banner advertisements have relied on explicit memory tests such as recognition and recall (Bayles, 2000; Drèze and Hussherr, 2003; Burke et al., 2005; Calisir and Karaali, 2008; Chatterjee, 2008). Explicit testing is limited in what it can tell us, not least because information that is presented in banner advertisements but seemingly ignored may still be processed to some extent, such that retained information may be detectable using implicit measures even when it is not revealed using explicit measures (Heath and Nairn, 2005; Yoo, 2007, 2008). Indeed, several studies have demonstrated implicit memory for advertising content in the absence of explicit recall or recognition, as evidenced through priming effects arising in indirect memory testing (e.g., Petre, 2005; Yoo, 2007). In the present experiment we deployed both explicit and implicit memory tests to measure the memorability of banner advertisements so as to counter any shortcomings arising from an exclusive reliance on traditional, explicit testing methods.

Based on the empirical research and theoretical perspectives reviewed above, four predictions were formulated in relation to our reported experiment:

- Vertical banners (located bottom-right) will promote increased attention to the whole advertisement relative to horizontal banners (located bottom-center), as well as enhanced memory for banner contents;
- Banner advertisements containing a face will show increased attention to the whole advertisement relative to banner advertisements where a face is absent;
- Banner advertisements containing a face with mutual gaze will show increased attention to the face compared to banner advertisements containing a face with averted gaze;
- Banner advertisements containing a face with averted gaze will show increased attention to—and memory for—the advertising text and the product compared to banner advertisements either containing a face with mutual gaze or no face.

It should be noted that no predictions were made relating to possible interactive effects on attention and memory arising from the combined influences of the banner type (vertical vs. horizontal) and face condition manipulations. We had no a priori reasons to motivate specific hypotheses regarding the likely presence of moderator effects given the limited existing research that has been pursued on these factors to date.

## Materials and methods

### Participants

The study involved 72 participants (36 male, 36 female) aged between 18 and 32 years (*M* = 22.9 years, *SD* = 1.53 years). Participants were undergraduate and postgraduate students at Lancaster University, UK, studying in a range of disciplines. Each participant received £8 for taking part and all had extensive experience of using the Internet for a period of at least 8 months prior to the study.

### Design

The experiment involved a 2 × 3 mixed within-between participants design. The within-participants factor was the banner type on the webpage, with two levels: vertical banner (located at the bottom-right) vs. horizontal banner (located at the bottom-center). The between-participants factor—referred to as face condition—had three levels: banner advertisements without a face (no face); banner advertisements containing a face with mutual gaze looking directly at the observer (mutual gaze); and banner advertisements containing a face with averted gaze looking at the advertised text and product (averted gaze).

The dependent variables in the eye-tracking part of the experiment were the average fixation duration and the total dwell time within three regions of interest (ROIs) located within the banner advertisements: faces (where these were present), advertised text and product. Note that total dwell time is the sum of all fixation durations within a particular ROI. Research has suggested that longer average fixation durations and longer total dwell times are both indicative of information being more attention demanding and engaging (Rayner, 1998; Poole and Ball, 2006; Holmqvist et al., 2011). For the memory phase of the study the dependent variable for the explicit memory test was the recognition score for presented brand names, whereas for the implicit memory test it was the word fragmentation completion score for aspects of the advertising text. Participants were randomly assigned to one of the three face conditions, with an equal number of participants and an equal gender split in each condition.

### Equipment

An ASL eye-tracker was used to record participants' eye movements whilst they performed a goal-directed browsing task. An infrared camera mounted below the computer screen was used to capture eye-movement data by recording the reflections from a participant's retina and cornea that arose from light being projected at the eyes from an infrared LED. These reflections were used to calibrate gaze positions on the screen (Duchowski, 2003; Poole and Ball, 2006). The experiment was controlled via a desktop computer.

### Fabricated webpages

**Figure 1:**
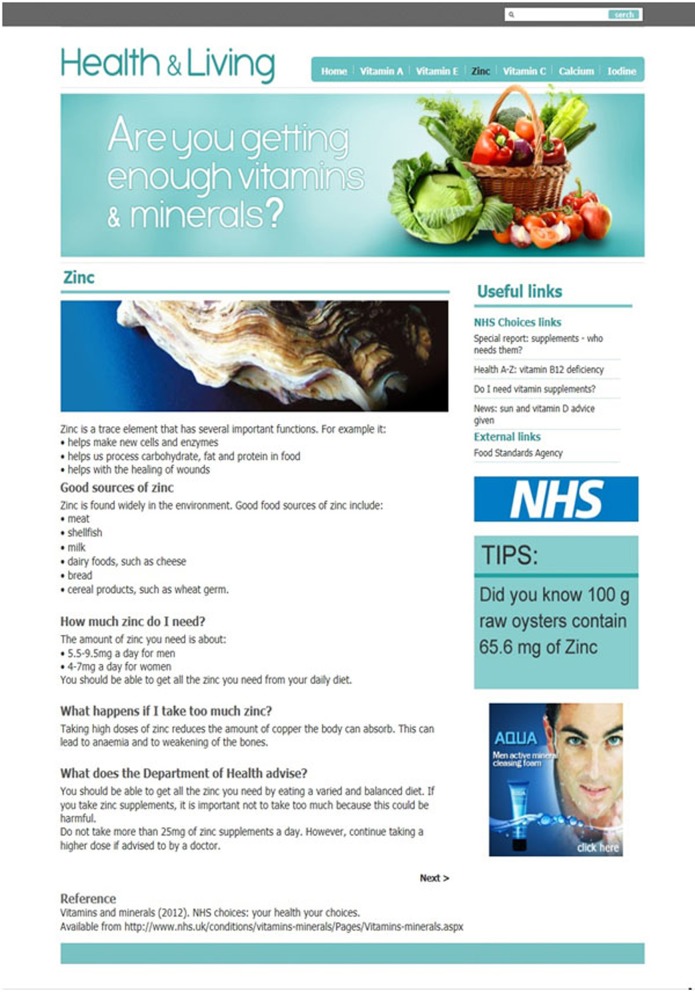
**An example webpage used in the experiment that presented information about zinc along with useful links and a vertical banner advertisement (located bottom-right)**.

**Figure 2:**
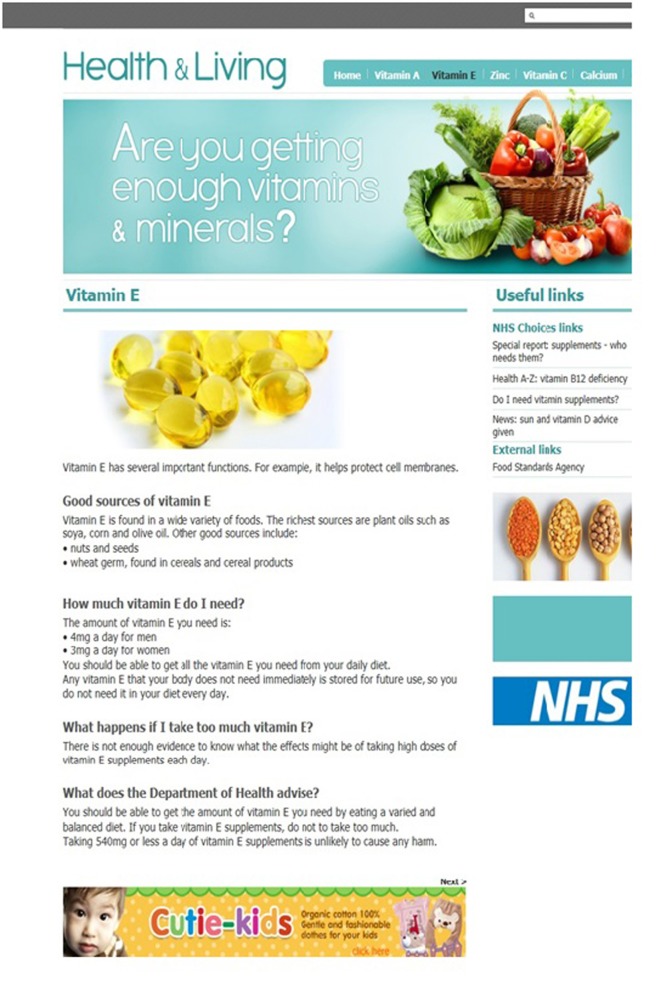
**An example webpage used in the experiment that presented information about Vitamin E along with useful links and a horizontal banner advertisement (located bottom-center)**.

Five thematically-linked webpages were designed that provided authentic factual information about healthy eating and the benefits of different vitamins and minerals (i.e., vitamin E, vitamin C, calcium, iodine, and zinc). One of the created webpages (i.e., concerning the mineral iodine) was always used as a “familiarization” trial so as to acquaint participants with the general style and information content of the webpages used in the experiment. The remaining four webpages were used as “target” trials, with two webpages presenting a vertical banner advertisement and two presenting a horizontal banner advertisement to each participant. The order of presentation of the target webpages was controlled in the manner explained in the Procedure section below. The fabricated webpages were realistic and in alignment with typical webpages that are found during everyday information searches on the Internet. Page headings, navigation bars, search boxes, and graphics were all located at conventional positions. Examples of two such pages are presented in Figures 1, 2. Note that Figure 1 depicts a vertical banner advertisement, whilst Figure 2 depicts a horizontal banner advertisement. All presented information on the webpages relating to vitamins and minerals was gender-neutral and was easy to understand. The information concerned good sources of particular vitamins and minerals, quantities needed for health benefits, side effects from excessive intake, Department of Health advice, useful links, and top tips.

### Banner advertisements

**Figure 3:**
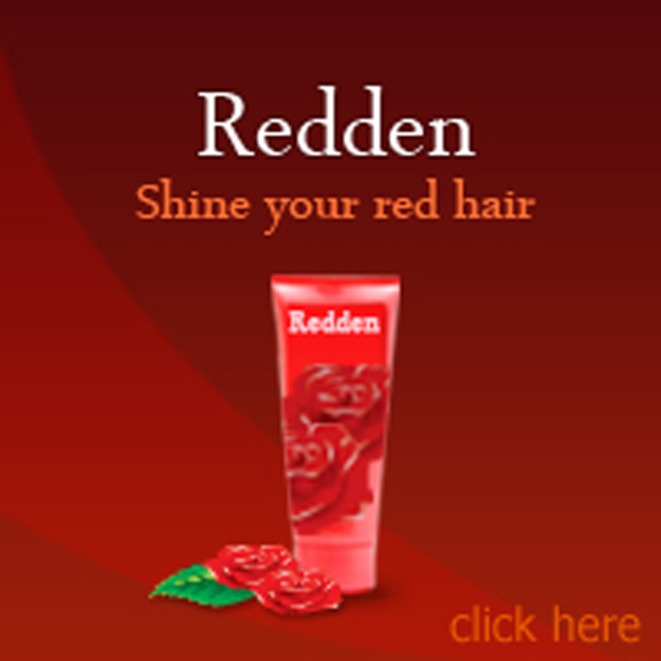
**Fabricated vertical banner advertisement for “Redden” in the no face condition**.

**Figure 4:**
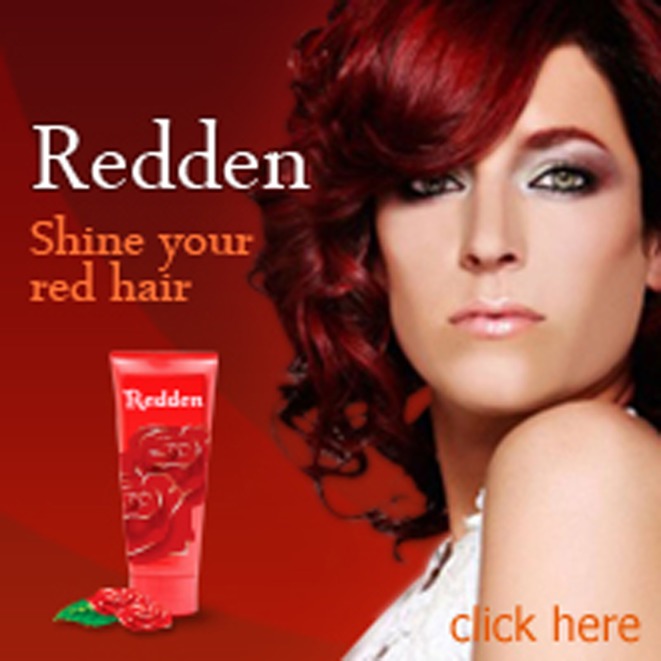
**Fabricated vertical banner advertisement for “Redden” in the mutual gaze condition**.

**Figure 5:**
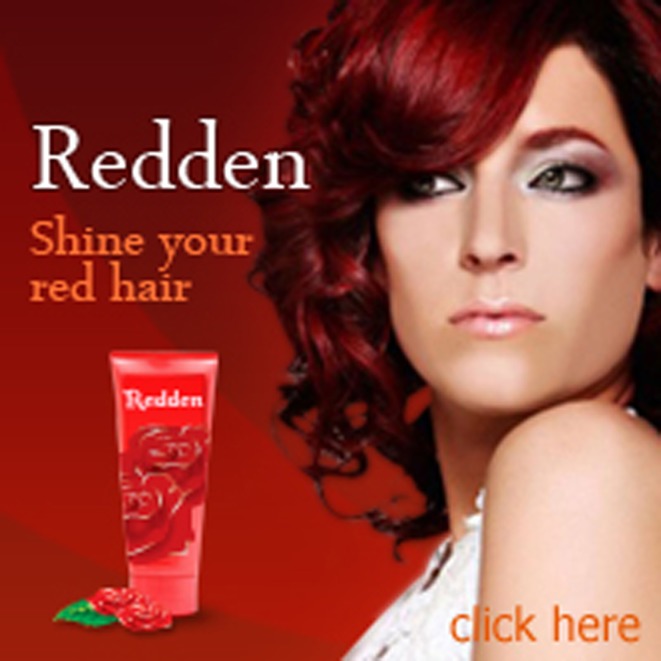
**Fabricated vertical banner advertisement for “Redden” in the averted gaze condition**.

**Figure 6:**
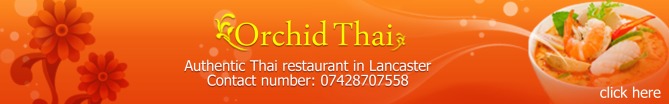
**Fabricated horizontal banner advertisement for “Orchid Thai” in the no face condition**.

**Figure 7:**
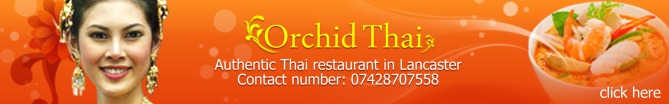
**Fabricated horizontal banner advertisement for “Orchid Thai” in the mutual gaze condition**.

**Figure 8:**
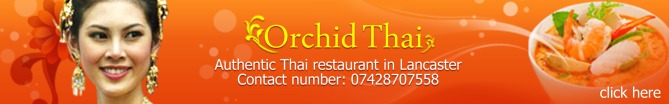
**Fabricated horizontal banner advertisement for “Orchid Thai” in the averted gaze condition**.

Vertical banner advertisements (226 × 246 pixels) were created for two fictitious products (i.e., “Redden” hair shine and “Aqua” mineral cleansing foam). Horizontal banner advertisements (606 × 96 pixels) were created for two other fictitious products (i.e., “Cutie-kids” clothing for children and “Orchid Thai” restaurant cuisine). All banner advertisements also included a small amount of product-specific text. Three versions of each banner advertisement were designed, one that did not include a face, one that included a face with the model's eyes looking straight ahead at the observer (mutual gaze), and one that included a face with the model's eyes averted toward the advertised text and product. Figures 3–5 show an example of a vertical banner advertisement for “Redden” hair-shine in each of the three conditions: no face (Figure 3), mutual gaze (Figure 4), and averted gaze (Figure 5). Figures 6–8 show an example of a horizontal banner advertisement for “Orchid Thai” restaurant cuisine across the same three face conditions.

To minimize the confounding effect of brand familiarity on attention and memory (e.g., Dahlén, 2001), all brands that we used were fictitious. In addition, we ensured that the product information in all banners was semantically incongruent with the information content of the webpage that they appeared on. We note that some research has shown that congruent advertisements increase attention to the advertising information and its subsequent memorability (e.g., Finlay et al., 2005; Hervet et al., 2011), whereas other research has revealed the opposite effect, whereby incongruent advertisements increase attentional capture and improve memory for advertising content (e.g., Dahlén et al., 2005). A recent eye-tracking study by Simola et al. (2013) that examined semantic incongruency in the context of newspaper advertisements revealed that incongruency increased attention to advertisements whereas congruency improved advert recognition. The key upshot of these conflicting findings is that it is vital to control for advertisement congruency/incongruency effects in a study such as the present one by standardizing the relationship between advertisements and webpage information. As noted, we achieved this by ensuring that all banner advertisements were semantically incongruent.

### Memory tests

To develop an explicit recognition test for the banner advertisements, two false lures were created for each advertisement by changing only the brand name. For example, the correct brand name for the “Orchid Thai” advertisement (see Figures 6–8) was replaced with either “Mung Mee” or “Oriental Cuisine.” In this way the other graphical and textual aspects of the advert were controlled so as to be consistent across the distractor items.

To assess people's implicit memory for the advertising message within each banner advertisement we developed a word fragment completion test in which participants were asked to complete fragments in which some consonants and vowels were missing (Fennis and Stroebe, 2010). To develop a list of word fragments we first constructed a pool of 35 words, with 20 being “target” words derived from the banner advertisements and 15 being “distractor” words selected from Tulving et al.'s (1982) study examining priming in word recognition. Having fragmented these words we then asked 30 students at Lancaster University (age range = 18–32 years, *M* = 22.6 years, *SD* = 1.06 years) to complete the fragments to make real words. Of the 35 words tested only 12 target words and 8 distractor words showed a correct completion rate of between 15 and 46%, which is a standard criterion for acceptability to avoid floor and ceiling effects (Tulving et al., 1982; Yoo, 2008). As examples, we note that the target words (and fragmented versions) for the banner advertisement relating to “Orchid Thai” restaurant cuisine, as shown in Figures 6–8, included: orchid (O_C_ _D), restaurant (R_ _ _ _ UR_ _T), and Lancaster (L_N_A_ _ _R). Examples of distracter items (and fragmented versions) included: mystery (_YS_ _RY), horizon (HO_ _ _ON), approval (APP_ _ _AL), and chimney (_ _IMN_Y).

The mean completion rates of the final 12 target words and 8 distractor words were 21.66% (*M* = 3.23, *SD* = 2.38) and 17.99% (*M* = 2.60, *SD* = 1.83), respectively. A paired-sample *t*-test demonstrated that there was no significant difference in word completion rate between the target and distractor words, *t*_(30)_ = 1.596, *p* = 0.121. Accordingly, these words were deemed to be suitable for use in the main experiment. We note that our word selection process meant that some banner advertisements were less well represented than others in the final implicit memory test used in our experiment. Indeed, the final test involved twice as many word fragments derived from the horizontal advertisements than from the vertical advertisements. To deal with this issue in our analysis of the implicit memory data (see below) we therefore derived “percentage correct” word fragment completion scores for items derived from vertical banners vs. horizontal banners, which standardized the scoring.

### Procedure

The experiment was run in a small, quiet eye-tracking laboratory. Participants were briefed and asked to sign a consent form prior to the study and were then asked to read information about a fictitious individual's symptoms of feeling unwell, as follows: “My name is Andy and I always get colds. I walk and move very slowly because my knees hurt. I cannot remember things well. Also, my skin is very dry, which makes me feel itchy and I easily get wounds.” Participants were subsequently instructed to browse through the five presented webpages so as to advise on the choice of vitamins and minerals suitable to relieve Andy's symptoms. This ensured that participants were provided with personal, goal-directed task instructions aimed at ensuring their focus on reading for comprehension. After this introductory session, but prior to browsing the webpages, participants were asked to sit about 50 cm from the computer screen and to undertake an eye-movement calibration procedure. This involved them fixating on nine small black crosses located in a 3 × 3 grid on the computer screen, without moving their head or body.

After calibration, participants were exposed to the initial familiarization webpage containing information about the mineral iodine in the absence of a banner advertisement. This trial aimed to acquaint participants with the style and content of the webpages, although it should be noted that participants were unaware that this trial served a purely practice function. Immediately after the familiarization trial participants were exposed to the four target webpages that formed the experimental trials, with each webpage presenting further information about vitamins and minerals in addition to either a vertical or horizontal banner advertisement. The order of these four experimental trials was counterbalanced such that there were 24 different orders per condition (i.e., 4!). This meant that each of the 24 participants within a condition received the target webpages in a unique order.

When the participant had finished reading the information about vitamins and minerals on a webpage they could move on to read the next webpage by clicking on the left button of the mouse. Throughout the webpage browsing task the eye tracker measured gaze behavior in relation to designated ROIs within the banner advertisement on each webpage, namely, the faces (where present), the brand name and associated text, and the product itself. Immediately after the browsing task each participant's implicit memory was tested using the word fragmentation completion test. This involved presenting participants with a sheet of paper that provided the list of incomplete words and asking them to complete them as best they could within 6 min. Participants then completed the recognition task, in which they were presented with two distractor advertisements and one target advertisement for each banner advertisement presented previously (the presentation order mapped onto the counterbalanced order in which banners had appeared during the browsing trials). The distractor advertisements were created by changing only the brand names from the target banner advertisements. Following the memory tests participants were asked to present a verbal account of how they would advise Andy in terms of his vitamin and mineral intake to improve his well-being.

## Results

### Eye-tracking data

The mean fixation duration data and the mean dwell time data were examined for skew and deviations from a normal distribution. It was found that all conditions showed a degree of positive skew—as is typical with time-based data—although in all cases but one the positive skew values were modest and less than +2.5, which is typically viewed as acceptable threshold for conducting parametric data analyses (e.g., see Tabachnick and Fidell, 1996). These violations of normality were confirmed through the application of the Kolmogorov-Smirnov test, which indicated that around 50% of the conditions involved data distributions that deviated significantly from normality.

Our approach to dealing with these modest violations from parametric testing assumptions was to pursue logarithmic transformations of our time-based data subsequent to the addition of a constant of 1.0 (to handle scores at or close to zero). This method was successful in reducing positive skew, normalizing the data and stabilizing variances. We next pursued equivalent parametric tests using both the transformed and the untransformed data. These separate analyses produced a very similar pattern of significant and non-significant effects, with similar effect magnitudes, although the transformed data typically yielded results with larger effect sizes. In the sub-sections below we limit our presentation of statistical findings to the outcomes of inferential tests undertaken on the *transformed* data. For ease of interpretation, however, we present graphical depictions of the untransformed time-based data in natural units (milliseconds).

We finally note that although we conducted a full set of inferential analyses for the mean fixation duration data *and* for the mean dwell time data, it was observed that both types of data produced near identical patterns of statistically significant effects. In order to limit the length of this article and provide a more focused narrative we only report below the results of the analyses undertaken on the mean dwell time data.

#### Mean dwell time on banner advertisements

**Figure 9:**
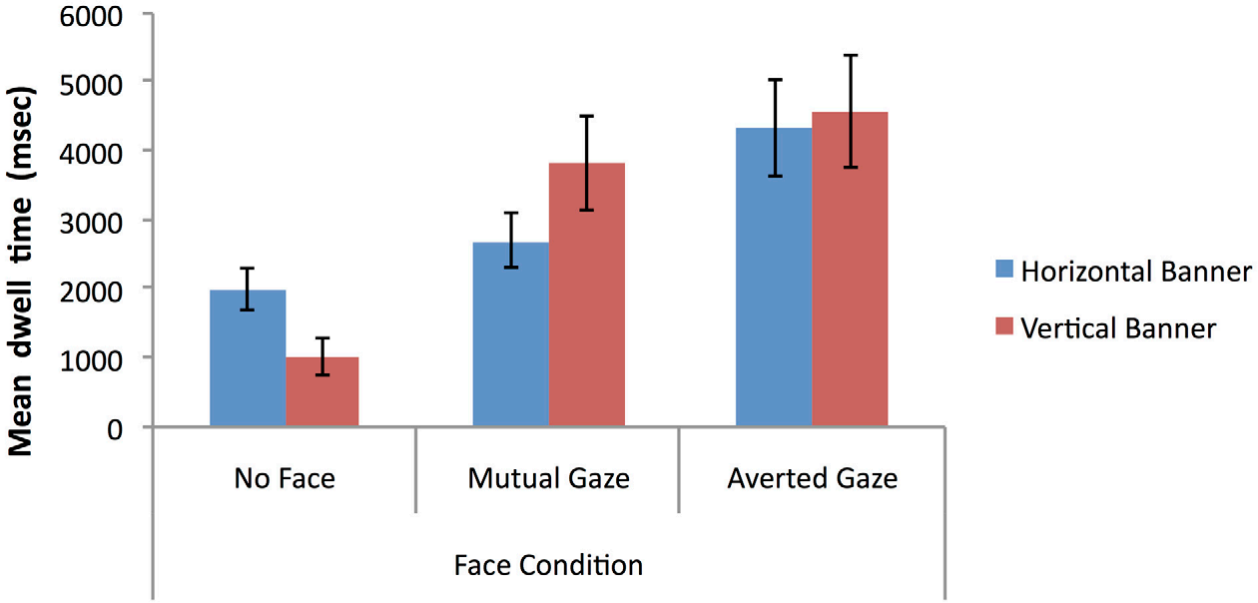
**Mean dwell time (milliseconds) on banner advertisements for vertical vs. horizontal banners as a function of face condition (error bars depict the standard error of the mean)**.

The first analysis of mean dwell time data examined the predictions that: (1) vertical banners (located bottom-right) give rise to increased attention to the whole advertisement relative to horizontal banners (located bottom-center); and (2) banner advertisements containing a face give rise to increased attention to the whole advertisement relative to banner advertisements where a face is absent. To test these predictions a 2 × 3 mixed factorial ANOVA was conducted on the log-transformed mean dwell time arising across the full extent of banner advertisements (vertical vs. horizontal) as a function of face condition (see Figure 9 for the natural data).

The analysis showed no significant main effect of banner type, *F* < 1, but did reveal a significant main effect of face condition, *F*_(2, 69)_ = 12.86, *p* < 0.001, η^2^_*p*_ = 0.27. *Post-hoc* Bonferroni tests (alpha level = 0.05) showed that the mean dwell time in the averted gaze condition and the mutual gaze condition were both significantly higher than in the no face condition, but there was no significant difference between the mutual gaze and averted gaze conditions. There was also a significant banner type × face condition interaction, *F*_(2, 69)_ = 4.01, *p* = 0.02, η^2^_*p*_ = 0.10, with vertical banners attracting more attention than horizontal banners in the mutual gaze condition, with the reverse being the case in the no face condition and with no difference in the averted gaze condition. Overall these findings do not provide any clear-cut support for increased attention arising for vertical banners over horizontal ones, but they do support the prediction that banner advertisements containing a face with either averted gaze or mutual can increase attentional capture relative to banner advertisements where a face is absent—at least when attentional capture is measured in terms of mean dwell time.

#### Mean dwell time on the region of interest relating to the face

Our next analysis of the mean dwell time data focused on the prediction that banner advertisements containing a face with mutual gaze give rise to increased attention to the face ROI compared to banner advertisements containing a face with averted gaze. To examine this prediction we conducted a 2 × 2 mixed factorial ANOVA with a within-participant factor of banner type (vertical vs. horizontal) and a between-participants factor of face condition (mutual gaze vs. averted gaze). Note that the reason for conducting a 2 × 2 ANOVA for this analysis compared to the 2 × 3 ANOVA in the previous analysis was simply a consequence of the fact that a face ROI did not exist in the banners that were employed in the no face condition. Such banners did not therefore include an ROI that could act as a meaningful comparison region to the face ROI that existed in the mutual gaze and averted gaze conditions.

**Figure 10:**
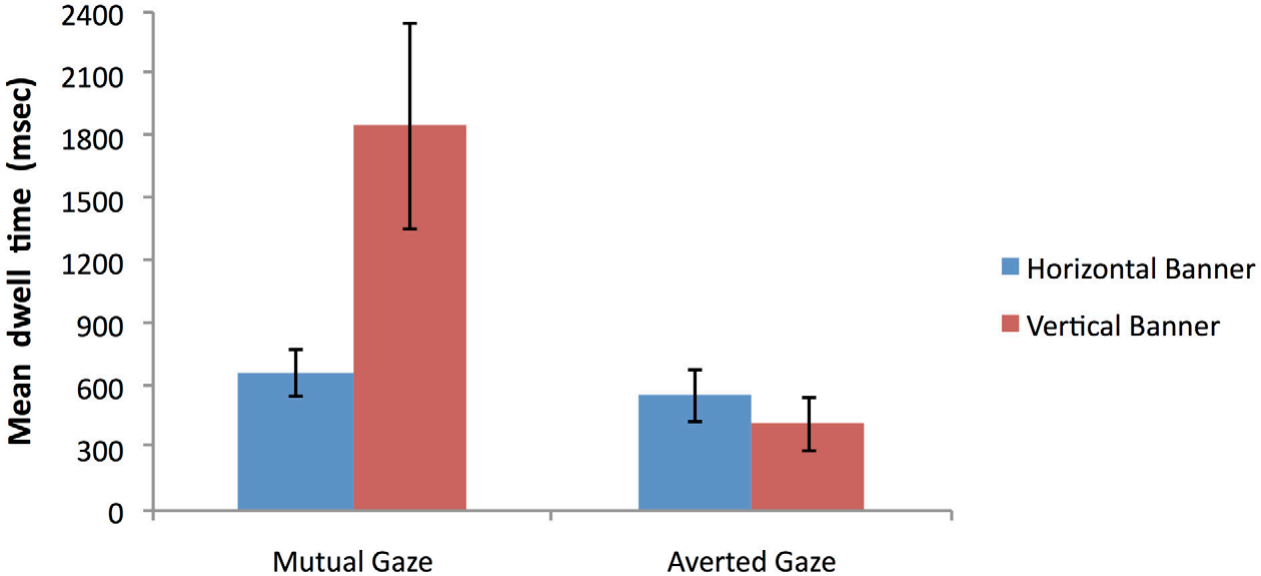
**Mean dwell time (milliseconds) on the face ROI for vertical vs. horizontal banner advertisements as a function of face condition (error bars depict the standard error of the mean)**.

The dependent variable in this analysis was the log-transformed mean dwell time on the face ROI (see Figure 10 for natural data). The main effect of banner type was not significant, *F*_(1, 46)_ = 2.57, *p* = 0.12, η^2^_*p*_ = 0.05. There was, however, a significant main effect of face condition, *F*_(1, 46)_ = 11.19, *p* = 0.002, η^2^_*p*_ = 0.20, with the mutual gaze condition promoting increased mean dwell time on faces relative to the averted gaze condition. There was also a significant banner type × face condition interaction, *F*_(1, 46)_ = 7.43, *p* = 0.009, η^2^_*p*_ = 0.14. Figure 10 shows that for the horizontal banners the mean dwell time on faces was similar whether the faces involved mutual or averted gaze. In contrast, for vertical banners the mean dwell time on faces was longer in the mutual gaze condition than the averted gaze condition, suggesting that vertical banners are more sensitive to the facial gaze manipulation than horizontal banners.

#### Mean dwell time on the regions of interest relating to the advertising text and product

**Figure 11:**
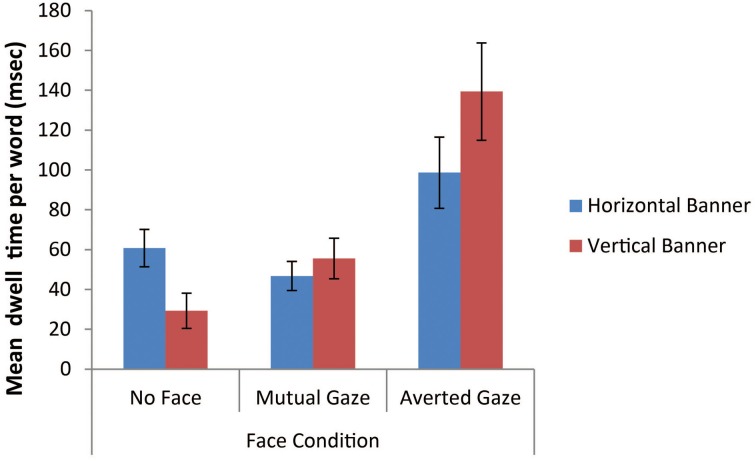
**Mean dwell time per word (milliseconds) within the advertising text ROI for vertical vs. horizontal banner advertisements as a function of face condition (error bars depict the standard error of the mean)**.

We next assessed the prediction that banner advertisements containing a face with averted gaze give rise to increased attention to the advertising text and the product compared to banner advertisements either containing a face with mutual gaze or no face. Our first analysis involved undertaking a 2 × 3 mixed factorial ANOVA to examine the log-transformed mean dwell time on *each word of advertising text* within vertical vs. horizontal banners across all three face conditions (see Figure 11 for natural data). We analyzed mean dwell time per word in order to control for the fact that there was twice as much text present in the horizontal banner advertisements (*M* = 11 words), than in the vertical banner advertisements (*M* = 5.5 words). To derive an approximation of a participant's mean dwell time per word for a particular banner we took their overall dwell time on the text ROI and divided this by the number of words within the ROI (see Ball et al., 2005, for another application of this “dwell time per word” standardization procedure).

The analysis of the resulting text-oriented dwell time data showed that there was no main effect of banner type, *F* < 1, but there was a significant main effect of face condition, *F*_(2, 69)_ = 12.24, *p* < 0.001, η^2^_*p*_ = 0.26. A series of *post-hoc* Bonferroni tests (alpha level = 0.05) were pursued to follow up the main effects of face condition. These tests revealed that the mean dwell time per word in the no face condition was not significantly different to that in the mutual gaze condition. However, both the no face condition and the mutual gaze condition had significantly lower mean dwell times per word than the averted gaze condition. The ANOVA also revealed a significant banner type × face condition interaction, *F*_(2, 69)_ = 5.38, *p* = 0.007, η^2^_*p*_ = 0.14. The data depicted in Figure 11 suggest that in the no face condition there was increased dwell time on the text of the horizontal banners compared to the vertical banners, whilst the pattern reversed in the averted gaze condition. In the mutual gaze condition there was little difference between horizontal and vertical banner in terms of dwell time on the advertising text.

**Figure 12:**
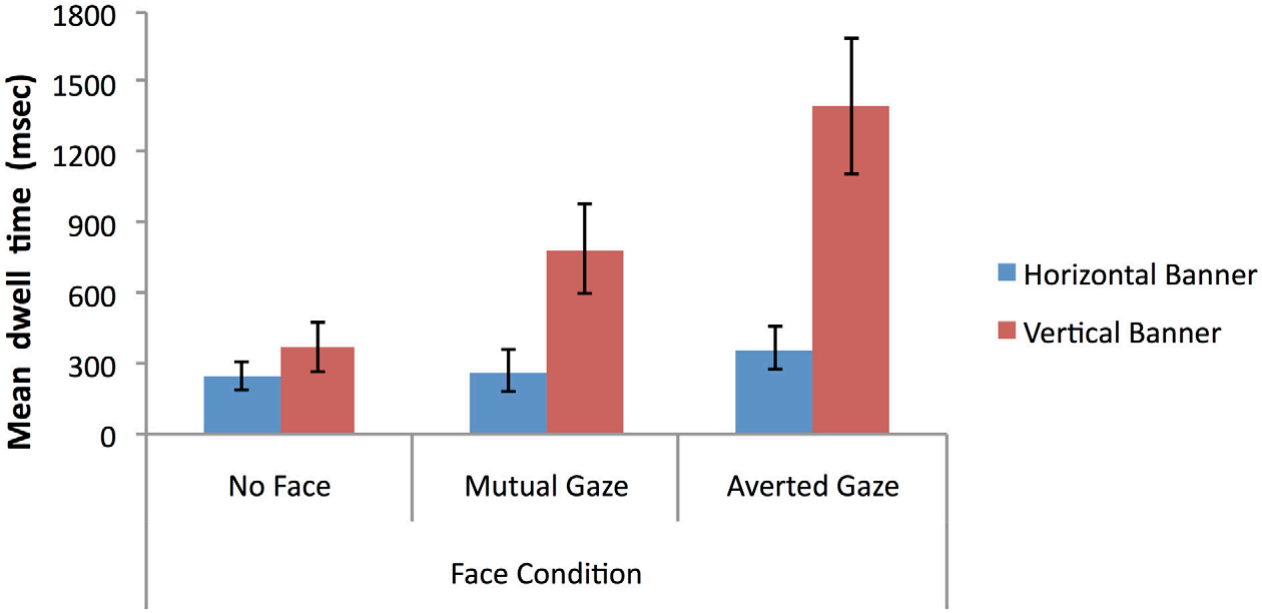
**Mean dwell time (milliseconds) on the product ROI for vertical vs. horizontal banner advertisements as a function of face condition (error bars depict the standard error of the mean)**.

The second ANOVA that we conducted examined log-transformed mean dwell time on the *product* ROI for vertical vs. horizontal banners across all three face conditions (see Figure 12 for natural data). This analysis revealed that there was a significant main effect of banner type, *F*_(1, 69)_ = 18.32, *p* < 0.001, η^2^_*p*_ = 0.21, with participants' mean dwell times on the product in the vertical banners being longer than in the horizontal banners. There was also a significant main effect of face condition, *F*_(2, 69)_ = 6.31, *p* = 0.003, η^2^_*p*_ = 0.16. A series of *post-hoc* Bonferroni tests (alpha level = 0.05) were pursued to follow up the main effects of face condition. It was observed that the mean dwell time on the product region of banner advertisements in the no face condition was not significantly different to the mutual gaze condition. There was also no significant dwell time difference on products in the mutual gaze vs. averted gaze conditions. However, the no face condition had a significantly lower mean dwell time on the product compared to the averted gaze condition. The ANOVA also gave rise to a significant banner type × face condition interaction, *F*_(2, 69)_ = 3.20, *p* = 0.047, η^2^_*p*_ = 0.09. This interaction effect appears to be caused by the incrementally increasing dwell time on product information in vertical banners that arises across the no face condition, followed by the mutual gaze condition, followed by the averted gaze condition. The horizontal banners show no such effect across face conditions.

### Memory data

#### Explicit memory for brand information

We predicted that that banner advertisements containing a face with averted gaze would show increased explicit memory for brand information compared to banner advertisements either containing a face with mutual gaze or no face. To examine this prediction we conducted a 2 × 3 mixed factorial ANOVA on correct recognition scores for brands for vertical vs. horizontal banners across all three face conditions (no face vs. mutual gaze vs. averted gaze). This ANOVA revealed no main effect of banner type, *F* < 1, but it did reveal a significant main effect of face condition, *F*_(2, 69)_ = 19.43, *p* < 0.001, η^2^_*p*_ = 0.36. The banner type × face condition interaction was not reliable, *F* < 1. The main effect of face condition was explored using *post-hoc* Bonferroni tests (alpha level = 0.05) and showed that the mean recognition score for brand names in the no face condition (0.54 items) was significantly lower than for products in the mutual gaze condition (1.06 items) and the averted gaze condition (1.50 items). The better recognition performance in the averted gaze condition compared to the mutual gaze condition was also shown to be statistically reliable. These results provide good support for the predicted increase in the recognition of product names in the averted gaze condition relative to the other conditions.

#### Implicit memory for the advertising text

We predicted that banner advertisements containing a face with averted gaze would show increased implicit memory for the advertising text compared to banner advertisements either containing a face with mutual gaze or no face. To test this prediction we conducted a 2 × 3 mixed factorial Analysis of Covariance (ANCOVA) on participants' implicit memory scores (i.e., their percentage correct word fragment completions for word items contained in vertical banners vs. word items contained in horizontal banners) across all three face conditions (no face vs. mutual gaze vs. averted gaze). This analysis included participants' correct *distracter item* word fragment completions as a covariate, since performance in relation to such distractor items that have not been encountered in the context of the experiment can be viewed as a good measure of a participant's baseline word fragment completion ability (see Ball et al., 2010).

**Figure 13:**
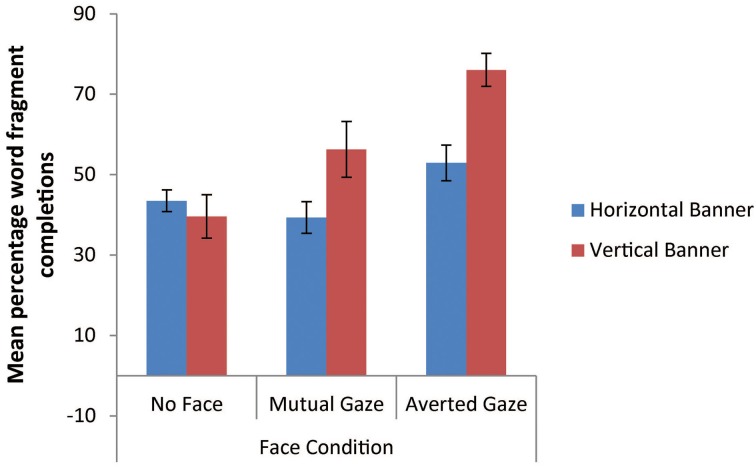
**Mean percentage correct word fragment completions for items as a function of banner type (vertical vs. horizontal) and face condition (error bars depict the standard error of the mean)**.

This ANCOVA analysis of the percentage of correct word fragment completions (Figure 13) revealed no main effect of banner type, *F*_(1, 68)_ = 2.71, *p* = 0.01, η^2^_*p*_ = 0.04, but it did give rise to a significant main effect of face condition, *F*_(2, 68)_ = 14.84, *p* < 0.001, η^2^_*p*_ = 0.30. The banner type × face condition interaction was also reliable, *F*_(2, 68)_ = 7.72, *p* = 0.001, η^2^_*p*_ = 0.19. The main effect of face condition was explored further using *post-hoc* Bonferroni tests (alpha level = 0.05), which indicated that the mean percentage target completion score in the mutual gaze condition (*M* = 47.79%) was not significantly different to the no face condition (*M* = 41.54%). However, the averted gaze condition was associated with a significantly higher mean target completion score (*M* = 64.48%) relative to each of the other two conditions. These results indicate that, as predicted, banner advertisements containing faces with averted gaze looking at advertised texts have a greater ability to improve participants' implicit memory performance for advertising contents than do banner advertisements containing faces with mutual gaze cues or advertisements with no faces.

This significant interaction between banner type and face condition appears to be caused by the more marked improvement in implicit memory scores for the vertical banners compared to the horizontal banners that arises across the no face condition, followed by the mutual gaze condition, followed by the averted gaze condition (see Figure 13). Again, this finding supports the eye tracking results reported above, which revealed that the vertical banners in our study were more sensitive to the facial gaze manipulation than were the horizontal banners. It appears, moreover, that such increased sensitivity to advertising contents is consequential, giving rise to enhance implicit memory performance for the adverting text in the banners that were attended to more assiduously, most notably the vertical banners containing faces with averted gaze.

## Discussion

The present study used a combination of eye-tracking analysis and explicit and implicit memory testing to determine the effects on attention to (and memory for) banner advertisements arising from two factors: (1) the location of banner advertisements on webpages (i.e., vertical banners positioned bottom-right vs. horizontal banners positioned bottom-center); and (2) the presence of facial images within banner advertisements (i.e., no face vs. face with mutual gaze vs. face with averted gaze).

In relation to the issue of the location of banner advertisements, it is noteworthy that some prior studies have suggested that the bottom-right and the bottom-center areas of webpages may typically be overlooked by web users (e.g., Nielsen, 2006; Djamasbi et al., 2010). However, more recent experimental research by Simola et al. (2011) has indicated that heightened levels of attentional capture can arise for vertical banner advertisements that are co-located alongside webpage text. The bottom-right located vertical banners that we examined in our experiment have not previously been examined in terms of attention and memory, but based on Simola et al.'s (2011) evidence we predicted that these would be associated with increased attentional capture and improved memory relative to the bottom-center located horizontal banner advertisements.

In relation to the issue of face presence within banner advertisements, the critical comparison in our study was between performance arising in a control condition in which the presented advertisement did not include a face vs. performance in experimental conditions in which the advertisement contained a face with mutual gaze (directed at the viewer) or a face with averted gaze (directed at the product and advertising text). Based on extant evidence (e.g., Straub, 2008), it was predicted that faces with mutual gaze would result in people paying more attention to the model's face rather than to the text or products in the advertisements. In contrast, faces with averted gaze were predicted to orient the focus of the viewer's attention automatically to the text or products embedded in the advertisements. Such increased attention to advertising information was also predicted to impact on the viewer's memory for brands and advertising messages. In our study we not only used an explicit recognition test to measure memory for brands, but also an implicit word fragment completion test to assess more subtle, priming-based evidence for the memorability of advertising information.

### The role of banner location in cueing attention to and memory for banner advertisements

The primary measure that we used to determine the attention that a participant paid to a banner advertisement was their dwell time on the banner, which could also be broken down further into the component dwell times on specific ROIs, including the face, text, and product. Based on Simola et al.'s (2011) research, we predicted that vertical banners (located bottom-right) would promote increased attention to the whole advertisement relative to horizontal banners (located bottom-center) by virtue of being co-located to the right of the text on the webpage. Contrary to this prediction our analyses indicated no main effect of banner type on the overall dwell time measure. The banner type factor was, however, found to interact with face condition, with vertical banners attracting more attention than horizontal banners in the mutual gaze condition, with the reverse being the case in the no face condition—and with no difference in the averted gaze condition. The fact that the overall attention-attracting capacity of banners is moderated by face condition affirmed the need to pursue more detailed dwell time analyses (discussed in the next sub-section) that focused on the way in which people's attention is distributed across specific ROIs within vertical and horizontal banners.

Our analyses also aimed to determine any influence of banner location on memory for banner contents. Our recognition measure of *explicit* memory for brand information showed neither a main effect of banner type nor an interaction between banner type and face condition. The absence of a banner type effect on recognition memory is unsurprising given the lack of any influence of this factor on the overall dwell time measure, as noted above. Our examination of the *implicit* memory measure (percentage correct word fragment completions for words that had appeared in vertical vs. horizontal banners) also revealed the absence of a main effect of banner type, supporting the explicit memory findings. The analysis did indicate, however, that the banner type factor interacted with face condition, with more manifest improvement in implicit memory scores for the vertical banners compared to the horizontal banners across the no face condition, mutual gaze condition and averted gaze condition, respectively. We suggest that these observations support the dwell time findings, which revealed that the vertical banners we used were more sensitive than the horizontal banners to facial gaze manipulation (see below for further discussion).

### The role of faces in cueing attention to banner advertisements

The overall dwell time data confirmed that the participants exposed to banners containing faces showed increased attention to the banner relative to banners where a face was absent. Having established the potency of faces to attract attention to banner advertisements our next series of analyses unpacked the effect of mutual gaze vs. averted gaze on viewers' attention to ROIs within the banner, such as the face itself and the text and product information.

In terms of the face ROI, we found that the mutual gaze condition led to substantially longer dwell times on the face itself compared to the averted gaze condition. This finding supports previous evidence demonstrating that mutual gaze has a unique capacity to capture a viewer's attention, leading to adverse consequences in terms of performance on a primary visual search task relating to the identification of a non-facial stimulus within the search array (e.g., Senju et al., 2005; Conty et al., 2006; Frischen et al., 2007). This analysis also revealed a significant banner type × face condition interaction, with the evidence indicating that vertical banners have greater potency than horizontal banners to attract increased attention to faces that involve mutual gaze as opposed to averted gaze. This increased attentional sensitivity to the specific contents of vertical banners provides some support for predictions that derived from Simola et al.'s (2011) research.

In terms of the text and product ROIs, our analyses gave rise to some further striking findings. In particular, it was evident that the averted gaze condition promoted significantly enhanced engagement with the advertising text (measured in terms of dwell time per word) and product information compared to either the mutual gaze condition or the no face control condition. This observation supports a key prediction concerning the power of averted gaze cues to orient attention by producing a reflexive shift in viewers' attention toward a specific item located in the direction of the gaze (cf. Ricciardelli et al., 2002; Mansfield et al., 2003). In an online advertising context it seems that once attention has been attracted toward text and product information as a result of a model's gaze cues then this can augment the possibility of the viewer actually engaging further in understanding (and potentially assimilating) the advertised brand and messages. The analysis of mean dwell time per word in relation to the advertising text also demonstrated an interaction between banner type and face condition. In particular, it was evident that in the no face condition there was increased dwell time on the text of the horizontal banners compared to the vertical banners, whilst the pattern reversed in the averted gaze condition.

In relation to the analysis that examined dwell times on the advertised product we observed a significant main effect of banner type, with mean dwell times on the product in the vertical banners being longer than in the horizontal banners. The increased attentional capture by the product contents of vertical banners relative to horizontal banners provides some further support for predictions that derived from Simola et al.'s (2011) research. The analysis of mean dwell times on the product information also gave rise to an interaction between banner type and face condition, whereby vertical banners located to the bottom-right of pages were differentially sensitive to the face condition manipulation relative to horizontal banners (located bottom-center), which showed little sensitivity to the face condition manipulation. The shortest dwell time on products in vertical banners arose in the no face condition, whilst the highest dwell time on products in vertical banners arose in the averted gaze condition (i.e., the natural data showed a near 5-fold increase in product dwell time). The vertical banners with mutual gaze were intermediate in terms of the dwell time on products. The horizontal banners showed no such effect across face conditions, receiving a low dwell time on product information in all conditions.

This latter evidence again supports the notion that vertical banners located to the right of webpage text are rather different in their attention-attracting capacity compared to horizontal banners that are located below webpage text (cf. Simola et al., 2011). Although, as noted, there was no evidence in our dataset that vertical banners attracted more *overall* attention than horizontal banners, it nevertheless appears that the pattern of attentional capture to vertical banners is highly sensitive to the facial cues that are present. In other words, it seems that once a person's attention had been gained by vertical banners then the subsequent distribution of attention is very much under the control of the embedded eye-gaze cues.

### The role of faces in enhancing memory for banner advertisements

The use of an explicit brand-recognition test revealed, as predicted, that participants were better at recognizing brand names that had been embedded in banner advertisements receiving the most attention on the relevant text and product information, that is, brand names in the averted gaze condition. To corroborate these relationships we report here the results of: (1) a correlation analysis examining the association between the mean dwell time on banner *text* and a participant's total brand recognition score; and (2) a correlation analysis examining the association between the mean dwell time on the *product* within the banner and a participant's total brand recognition score. The respective correlations were significant and indicated the existence of the predicted positive association (*r* = 0.272, *p* = 0.021; *r* = 0.400, *p* = 0.001; both tests two-tailed). These findings are consistent with a range of evidence regarding attention and memory, suggesting that items that are attended to (as determined by eye-tracking data) are subsequently remembered (e.g., Irwin and Zelinsky, 2002), and that the longer the time spent viewing an item, then the greater the ability to remember it (e.g., Zelinsky and Loschky, 2005).

The use of an implicit memory test (i.e., indirect priming in a word fragment completion task) likewise supported the prediction that participants would be better at showing retention of aspects of the advertising message that had been embedded in banner advertisements receiving the most attention on the advertising text and product information, that is, the text and products in the averted gaze condition. To corroborate these relationships (as in the case of the explicit memory test noted above) we report here the results of: (1) a correlation analysis examining the association between the mean dwell time on banner *text* and a participant's percentage correct word fragment completion score; and (2) a correlation analysis examining the association between the mean dwell time on the banner *product* and a participant's percentage correct word fragment completion score. The respective correlations were positive and significant and supported the presence of the predicted association (*r* = 0.371, *p* = 0.001; *r* = 0.376, *p* = 0.002; both tests two-tailed). We note that Yoo (2008) reported a similar implicit memory effect for banner advertisements, suggesting that an increase in attention in terms of consciously processing web advertisements could enhance implicit memory performance in terms of remembering the advertising words embedded in those advertisements. In sum, our findings suggest that successful implicit memory performance in remembering advertising messages in banner advertisements is critically related to the high level of attention being paid to those messages.

### Implications and future work

The present findings go a step beyond previous research on banner advertising by providing a demonstration that embedding faces with averted gaze within online banner advertisements can not only capture web users' attention while they are searching for information, but can also specifically increase their attention to the advertising message as well as brand details and product information. Furthermore, this research reveals that this increased attention to advertising information is consequential, that is, it leads to the enhanced assimilation of such information, promoting a significantly increased ability to remember the content of advertisements, as determined by means of explicit and implicit memory testing. These attentional and memorial effects can occur whether faces with averted gaze are placed in vertical banners located at the bottom-right of webpages or horizontal banners located at the bottom-center of webpages.

Our findings suggest that that it is possible for advertisers to design graphical banner advertisements with embedded faces in ways that have considerable potency to orient a viewer's eye gaze toward key contents within the banner that pertain to advertising messages, brand information and product details. Vertical banners located at the bottom-right of webpages show particular sensitivity to facial manipulations involving gaze cues, with averted gaze leading, for example, to increased attention on product information as opposed to the face itself. Taken from a different perspective, the present results also attest to what are problematic designs to use for banner advertisements. For example, banners containing faces depicting mutual gaze are likely to invoke increased attention to the face at the expense of the viewer attending to advertising information. Particularly problematic are faces with mutual gaze within vertical banners located at the bottom-right of webpages, which engender especially intensive user engagement with the face itself rather than with advertising information.

As with many studies of online advertising using realistic stimuli there are limitations inherent in the present experiment that may provide an impetus for future research. First, although our use of fabricated banner advertisements with fictitious brand names reduced the potentially confounding effects of extraneous variables, such as product familiarity, we recognize that our findings may not necessarily generalize to real banner advertisements embedded in genuine website pages. It is, therefore, important for future research to explore how facial images with gaze cues have an effect on attention and memory in professional banner advertisements embedded in Internet pages so as to increase the external validity of our findings.

Second, the sizes of the vertical banners (226 × 246 pixels) and the horizontal banners (606 × 96 pixels) that we used were based on how well they fitted in the bottom-center location (in the horizontal axis) and the bottom-right location (in the vertical axis) on our constructed webpages. Although the use of these specific banner sizes allowed for an effective manipulation of banners within a naturalistic browsing context, it would be valuable for future research to examine more fully and systematically the impact of manipulating banner size across a range of dimensions. In this respect a follow-on study would also be worth conducting that carefully standardized the size (and also the content) of the vertical and horizontal banners so as to eliminate any potential confounds arising from a lack of control in these respects. We are especially conscious of the potential problems with data interpretation that can derive from a failure to control adequately for banner content across vertical vs. horizontal banner types. We know from previous research, for example, that low-level visual features are highly influential in directing attention in a bottom-up manner toward salient, localized areas of scenes and images (Theeuwes, 1994; Rayner, 1998). Indeed, contemporary models of visual attention typically include the concept of a “saliency map,” which is a theoretical construct that functions to integrate information across different low-level features within a scene (e.g., color, intensity, orientation) to form a unitary map that encodes the visual saliency of those features (e.g., see Itti and Koch, 2000). The “maximum” of the saliency map corresponds to the most salient location within the image or scene, which is believed to be the location that is most likely to attract visual attention (see Simola et al., for a relevant discussion of these concepts in an advertising context).

In these latter respects we concede that in our study there remained a possibility that salient low-level features within the advertisements that we used might have inadvertently been confounded with our banner type manipulation, despite our careful attempt to standardize banners in terms of information content relating to faces, products and text. Follow-up research could control for this issue more effectively by first applying a saliency algorithm to different advertisements so as to check their comparability in terms of their inherent featural saliency. Alternatively, an experimental design in which advertising content was systematically rotated across banner types and locations would also be a good way to help control for any influence of bottom-up feature salience on attention.

Third, the present study did not investigate data relating to the number of fixations on ROIs within banner advertisements, yet previous research has suggested that a large number of fixation counts on a particular area is indicative of the informativeness and importance of that area for viewers (e.g., Bojko, 2005). Hence, it would be useful for future research to analyse fixation counts on ROIs so as to inform an understanding of how facial images and gaze cues within banner advertisements impact the perceived importance of advertising information. In addition, the analysis of data relating to the duration of first fixations (e.g., Henderson and Hollingworth, 1999) might be useful so as to obtain further insights regarding the attention-grabbing capacity of faces depicting mutual or averted gaze cues in relation to different ROIs.

Finally, it is important for future research to investigate the effects of banner advertisements containing facial images on attention and memory in terms of sex differences. This is because research by Bayliss et al. (2005) has revealed intriguing evidence that females have a greater ability to encode gaze direction than males, such that the unique capacity that gaze cues have for the reflexive orientation of attention may be more pronounced for female than male viewers of web-based advertising information.

## Concluding remarks

The present findings demonstrate how facial images with averted gaze that are embedded within online banner advertisements provide powerful orienting cues that can increase web users' attention to advertising information that is incidental to their current, goal-directed search task. Importantly, this increased attentional engagement with advertising information manifests itself in an enhanced ability to remember advertising contents such as brand information and words linked to advertising messages. The study also demonstrates that the converse results arise when banner advertisements include embedded faces with mutual gaze. In this latter case although web users are attracted to attend to the banner advertisement, they engage disproportionately with the face itself at the expense of attending to advertising information, which generally limits any memory benefits that arise for brand information or adverting details. This detrimental impact of mutual gaze on attention to advertising products is particularly marked for vertical banners located at the bottom-right of webpages, whereas averted gaze cues in such banners have a positive impact on attention to product information. Our findings give good grounds for suggesting that advertisers could capitalize on the inclusion of averted gaze cues within online advertisements so as to enhance people's engagement with (and memory for) advertising messages, brand information and product details.

### Conflict of interest statement

The authors declare that the research was conducted in the absence of any commercial or financial relationships that could be construed as a potential conflict of interest.
